# Heterozygous *COL4A3* Variants in Histologically Diagnosed Focal Segmental Glomerulosclerosis

**DOI:** 10.3389/fped.2018.00171

**Published:** 2018-06-12

**Authors:** Matthias C. Braunisch, Maike Büttner-Herold, Roman Günthner, Robin Satanovskij, Korbinian M. Riedhammer, Pierre-Maurice Herr, Hanns-Georg Klein, Dagmar Wahl, Claudius Küchle, Lutz Renders, Uwe Heemann, Christoph Schmaderer, Julia Hoefele

**Affiliations:** ^1^Department of Nephrology, Klinikum Rechts der Isar, Technical University of Munich, Munich, Germany; ^2^Institute of Human Genetics, Klinikum Rechts der Isar, Technical University of Munich, Munich, Germany; ^3^Department of Nephropathology, Institute of Pathology, Friedrich-Alexander-University, Erlangen, Germany; ^4^Center for Human Genetics and Laboratory Diagnostics Dr. Klein, Dr. Rost and Colleagues, Martinsried, Germany

**Keywords:** Alport syndrome, *COL4A3*, focal segmental glomerulosclerosis, FSGS, nephrotic syndrome, hearing impairment

## Abstract

**Introduction:** Steroid-resistant nephrotic syndrome (SRNS) is one of the most frequent causes for chronic kidney disease in childhood. In ~30% of these cases a genetic cause can be identified. The histological finding in SRNS is often focal segmental glomerulosclerosis (FSGS). In rare cases, however, pathogenic variants in genes associated with Alport syndrome can be identified in patients with the histological finding of FSGS.

**Materials and Methods:** Clinical information was collected out of clinical reports and medical history. Focused molecular genetic analysis included sequencing of *COL4A5* and *COL4A3* in the index patient. Segregation analysis of identified variants was performed in the parents and children of the index patient.

**Results:** The female index patient developed mild proteinuria and microscopic hematuria in childhood (12 years of age). The histological examination of the kidney biopsies performed at the age of 21, 28, and 32 years showed findings partly compatible with FSGS. However, immunosuppressive treatment of the index patient did not lead to a sufficient reduction of in part nephrotic-range proteinuria. After the patient developed hearing impairment at the age of 34 years and her daughter was diagnosed with microscopic hematuria at the age of 6 years, re-examination of the index's kidney biopsies by electron microscopy revealed textural changes of glomerular basement membrane compatible with Alport syndrome. Molecular genetic analysis identified two missense variants in *COL4A3* in a compound heterozygous state with maternal and paternal inheritance. One of them is a novel variant that was also found in the 6 year old daughter of the index patient who presented with microscopic hematuria.

**Discussion:** We were able to show that a novel variant combined with a previously described variant in compound heterozygous state resulted in a phenotype that was histologically associated with FSGS. Molecular genetic analysis therefore can be essential to solve difficult cases that show an unusual appearance and therefore improve diagnostic accuracy. Additionally, unnecessary and inefficient treatment with multiple side effects can be avoided.

## Introduction

Steroid-resistant nephrotic syndrome (SRNS) is one of the most frequent causes for chronic kidney disease especially in young adulthood (1, 2). In around 30% of cases a monogenetic cause can be identified (3). Histological findings in SRNS largely present as focal segmental glomerulosclerosis (FSGS) with progressive glomerular scarring (4). To date, more than 30 genes associated with SRNS have been identified placing the podocyte into the center of attention regarding the pathogenesis of SRNS and FSGS (5). Immunosuppressive treatment of proteinuria in hereditary FSGS often cannot induce remission and is also poorly tolerated (6, 7).

In rare cases, FSGS can be caused by variants in *COL4A3* and *COL4A4*, both genes associated with Alport syndrome (AS) (8). AS typically occurs with the leading symptoms of progressive renal failure, sometimes associated with the extrarenal manifestations of hearing impairment (sensorineural deafness) and/or ocular abnormalities (anterior lenticonus, cataract) (9). In AS the first sign is microscopic hematuria in early childhood. Proteinuria increases as disease progresses. However, proteinuria commonly does not reach nephrotic range in contrast to FSGS or SRNS (10).

Variants in *COL4A5* are the most frequent causes involved in the pathogenesis of AS and account for about 85% of cases (9). Due to the X-linked inheritance of *COL4A5* mostly men are affected. In fewer cases, variants in *COL4A3* and *COL4A4* located at the long arm of chromosome 2 account for an autosomal recessive (14% of cases) or autosomal dominant (1% of cases) AS (11, 12). Male and female patients are equally affected.

Due to mainly economic reasons, genetic testing is often reluctantly used in clinical routine. Molecular genetic diagnosis can improve accuracy of disease classification, especially in phenotypes with multiple symptoms and unusual appearance and help to create personalized treatment options.

Here we report one novel *COL4A3* variant in a compound heterozygous state in a 34 year old woman with hematuria and proteinuria who presented initially with histological findings compatible with FSGS. Only after hearing impairment occurred later in life, genetic testing of AS associated genes was initiated and the correct diagnosis was made.

## Materials and methods

### Clinical case information

The study was approved by the local Ethics Committee of the Technical University of Munich and performed according to the standard of the Helsinki Declaration of 1975. Written informed consent was obtained from the index patient and their relatives for publication. Clinical and phenotype information was gathered out of clinical reports and medical history.

### Histology

Histological examinations of the kidney biopsies at the age of 28 and 32 years were performed with light microscopy of sections stained with Periodic acid-Schiff (PAS) reaction and hematoxylin and eosin staining, immunohistochemistry and electron microscopy. Immunohistochemistry of the latest biopsy was performed with antibodies specific for IgA, IgG, IgM, C1q, and C3c (all polyclonal rabbit antibodies, Dako; Dilution and Code No. IgA 1:200000, A0262, IgG 1:150000, A0423, IgM 1:100000, A0425, C1q 1:100000, A0136, C3c 1:100000, A0062) after digestion with PronaseE for 45 min. For detection Envision Kit (Dako) was applied and DAB was used as a chromogen. For electron microscopy fixed renal biopsies were dehydrated and embedded in Epon. Semithin and ultrathin sections were prepared and stained with methylene blue or uranyl acetate/lead citrate, respectively. Ultrathin sections were then analyzed with a Zeiss electron microscope LEO EM 910 or LEO EM 912 (Zeiss, Oberkochen, Germany) at various magnifications. Histological and electron microscopic images were only available from the latest kidney biopsy.

### Genetics

Blood samples were collected after written informed consent. Genomic DNA was extracted from peripheral blood of the index patient, her parents, and her children using the Gentra Puregene Blood Kit (Qiagen, Hilden, Germany) according to manufacturer's instructions. In the index patient exon 1 to 51 of *COL4A5* were examined followed by exon 1 to 52 of *COL4A3* using direct DNA sequencing on both strands applying the dideoxy chain termination method on an ABI capillary sequencer 3730 (Applied Biosystems, Foster City, USA). Primers were designed by Primer3 program (http://frodo.wi.mit.edu/primer3/input.htm). For segregation analysis, subsequent targeted sequencing was performed in both parents and children of the index patient in exon 26 and 52 of the *COL4A3* gene. DNA alignment and sequence variant analysis were carried out using the Sequence Pilot^CE^ software (JSI Medical Systems GmbH, Kippenheim, Germany) and compared to EMBL (European Molecular Biology Laboratory) and GenBank databases as well as our in-house database. All variants were validated in a second independent DNA sample. Scaled gene structure was created with the Gene Structure Display Server version 2.0 (13).

## Results

### Clinical phenotype

#### Index patient

**Figure 1:**
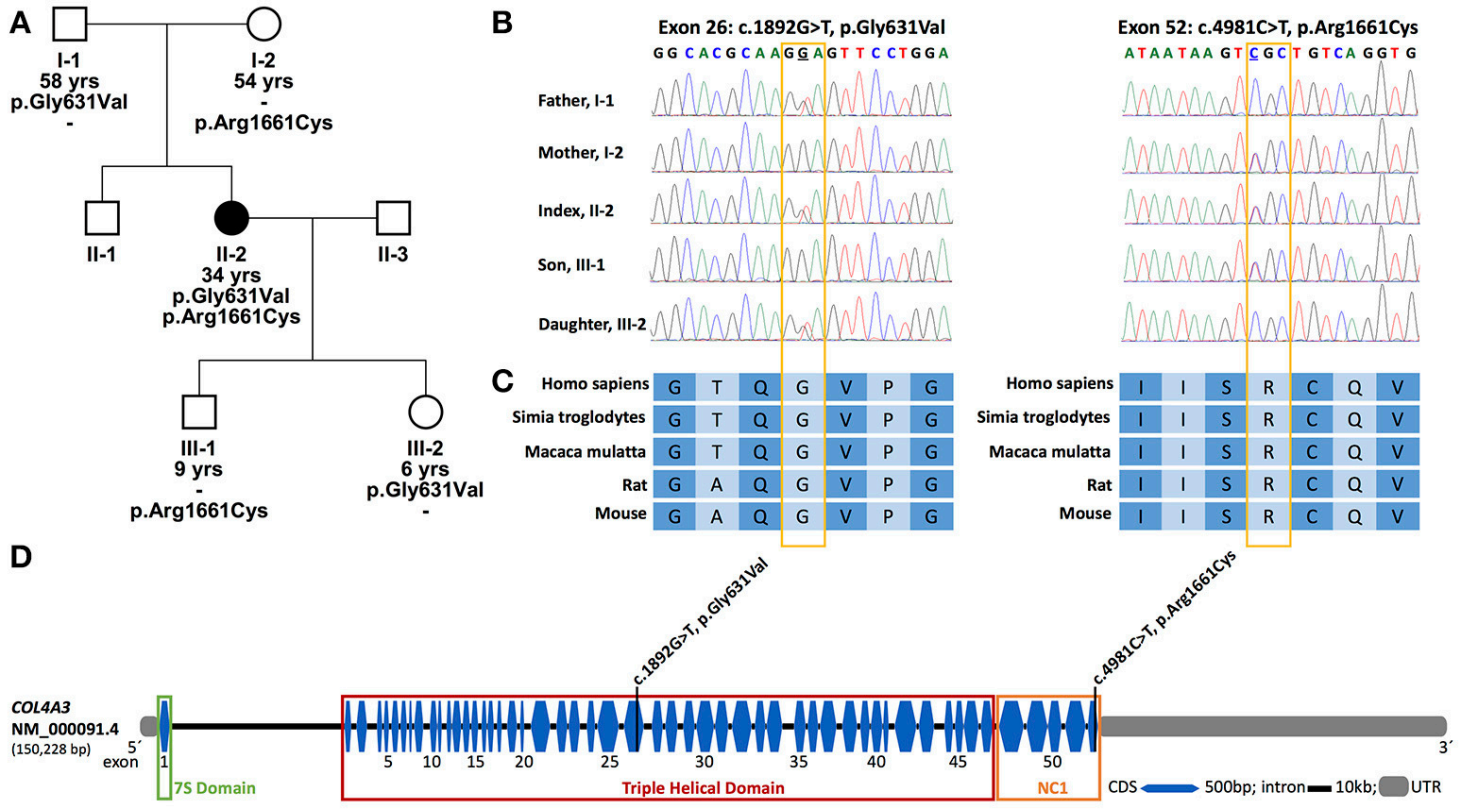
Pedigree of the family, conservations and location of identified variants. **(A)** Pedigree of the family; solid symbols, affected individuals; circles, females; squares, males; yrs, years. **(B)** Partial nucleotide sequence of exon 26 and 52 of *COL4A3* of the patient, her parents and her children. **(C)** Conservation of the affected amino acid over five different species; A, Alanine; C, Cysteine; G, Glycine; I, Isoleucine; P, Proline; Q, Glutamine; R, Arginine; S, Serine; T, Threonine; V, Valine. **(D)** Exon-intron structure of *COL4A3* (NM_000091.4) with the localization of the two identified variants. The variant p.Gly631Val lies in the central triple helical domain, whereas the variant p.Arg1661Cys lies in the C-terminal cysteine-rich non-collagenous domain (NC1) which is together with the 7S domain at the N-terminal a cross-linking domain. bp, base pairs; CDS, coding sequence; kb, kilo base pairs; UTR, untranslated region.

A 34 year old Caucasian woman of German ancestry presented in our department (Figure 1A, II-2). At the age of 12 years, mild proteinuria and microscopic hematuria was detected. This finding was, however, not followed.

At the age of 21 years, the index patient had, for the first-time, increased proteinuria (>1 g per day) and mild hypertension. The histology of a kidney biopsy showed, besides minimal mesangial cell proliferation, uncharacteristic findings. Therefore, no specific therapy was initiated. Unfortunately, no detailed report of the first kidney biopsy was available.

In the presence of persistent proteinuria of >1 g/d and a decrease of creatinine clearance from 83 to 65 mL/min/1.73 m^2^ re-biopsy of the kidney was performed at the age of 28 years. The diagnosis at that time was chronic kidney disease due to FSGS with secondary hypertension and proteinuria. The clinical presentation was normal, besides a slightly increased weight (body mass index 24.7 kg/m^2^) possibly due to the discrete lower leg edema. Especially, there was no report of hearing impairment. Further laboratory work-up is shown in Table 1. Ultrasound examination showed morphologically normal kidneys. The histology of the kidney biopsy revealed focal global glomerulosclerosis with mild chronic tubulo-interstitial damage. Electron microscopy showed one scarred glomerulus and one glomerulus with clumsy podocyte foot processes with effacement compatible with the diagnosis of primary FSGS. Due to a gross proteinuria and the progressive decline of kidney function an angiotensin-converting enzyme (ACE) inhibitor and immunosuppressive therapy with cyclosporine for 7 months and prednisolone were initiated.

**Table 1:** Abnormal blood and urine laboratory values.

	**Reference range**	**Values**	
		**28 years of age**	**32 years of age**
**BLOOD**			
Blood urea nitrogen	7–18 md/dL	19.6	39
Creatinine	0.5–1.1 mg/dL	1.2	1.1–1.4
Phosphate	2.5–4.5 mg/dL	–	4.7
GFR (MDRD)	>60 mL/min/1.73 m^2^	–	>60
Uric acid	3–7 mg/dL	8.1	–
Total serum protein	6.0–8.0 g/dL	5.6	5.7
Lactate dehydrogenase	<244 U/L	284	353–433
Anti-nuclear antibodies	<1: 120	1:120	–
**SERUM ELECTROPHORESIS**			
Alpha-1	1.5–4.0%	4.2	–
Alpha-2	5.0–9.0%	11.5	–
ß-globulin	7.0–12.0%	12.9	–
**URINE**			
Proteinuria	<150 mg/g creatinine	1465	316
Albuminuria	<20 mg/g creatinine	1384	–
Alpha-1-microglobulinuria	<14 mg/g creatinine	14.1	–
Leukocyturia	0–3 per visual field	–	4–10
Erythrocyturia	0–3 per visual field	-	4–10

*GFR, glomerular filtration rate; MDRD, Modification of Diet in Renal Disease*.

Four years later, at the age of 32 years, the patient experienced nephrotic range proteinuria (4.5 g per day). In the beginning, the patient responded well to immunosuppressive treatment with prednisolone (starting dose 60 mg, tapered to 7.5 mg once daily) and mycophenolate mofetil (MMF) (250 mg twice daily) with a reduction of the proteinuria to 1.7 g per day. To further reduce the proteinuria, MMF was increased to 500 mg twice daily. Nonetheless, proteinuria increased again to 4.3 g/d. Therefore, MMF was discontinued and the patient was admitted to our hospital for a kidney biopsy. Clinical presentation at that time was age-appropriate, besides discrete lower leg edema. Abnormal laboratory values are shown in Table 1.

**Figure 2:**
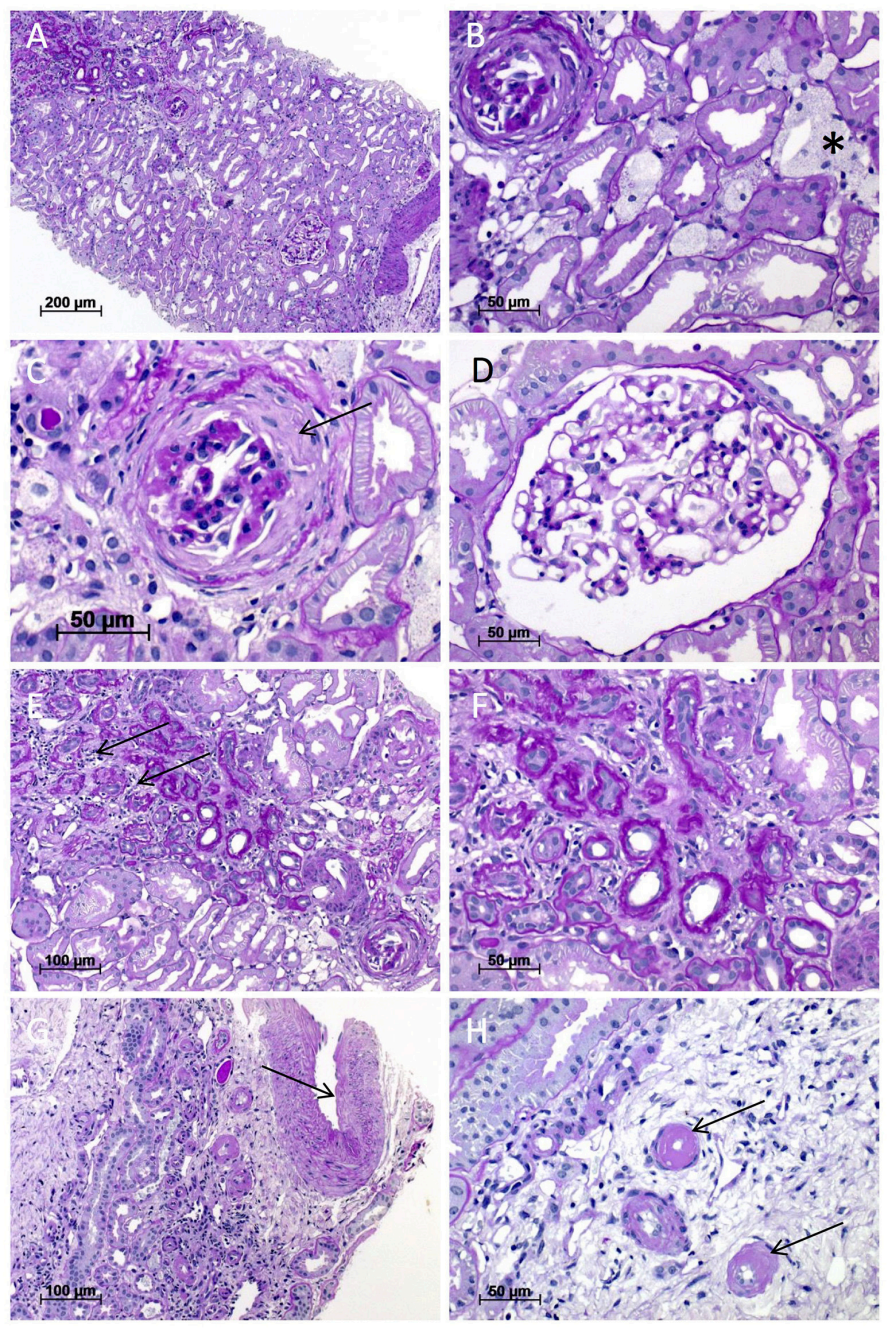
Light microscopy of the kidney biopsy. **(A)** Renal biopsy performed at the age of 32 years with patchy tubular atrophy and interstitial fibrosis (upper left; magnification x100). **(B)** Multiple interstitial foam cell nests (asterisk) and an obliterated glomerulus (upper left; magnification x400), which shows in **(C)** residues of fibrous synechia (arrow; magnification x400). **(D)** One unremarkable glomerulus (magnification x400). **(E,F)** Interstitial fibrosis and tubular atrophy with mild lymphoplasmacytic interstitial infiltrate (arrows) (**E**; magnification x200; **F**; magnification x400). **(G)** Arterial vessel walls showed mild thickening (arrow) and moderate arteriolar hyalinosis **(H)**. All images were taken from sections stained with Periodic acid-Schiff (PAS) reaction.

**Figure 3:**
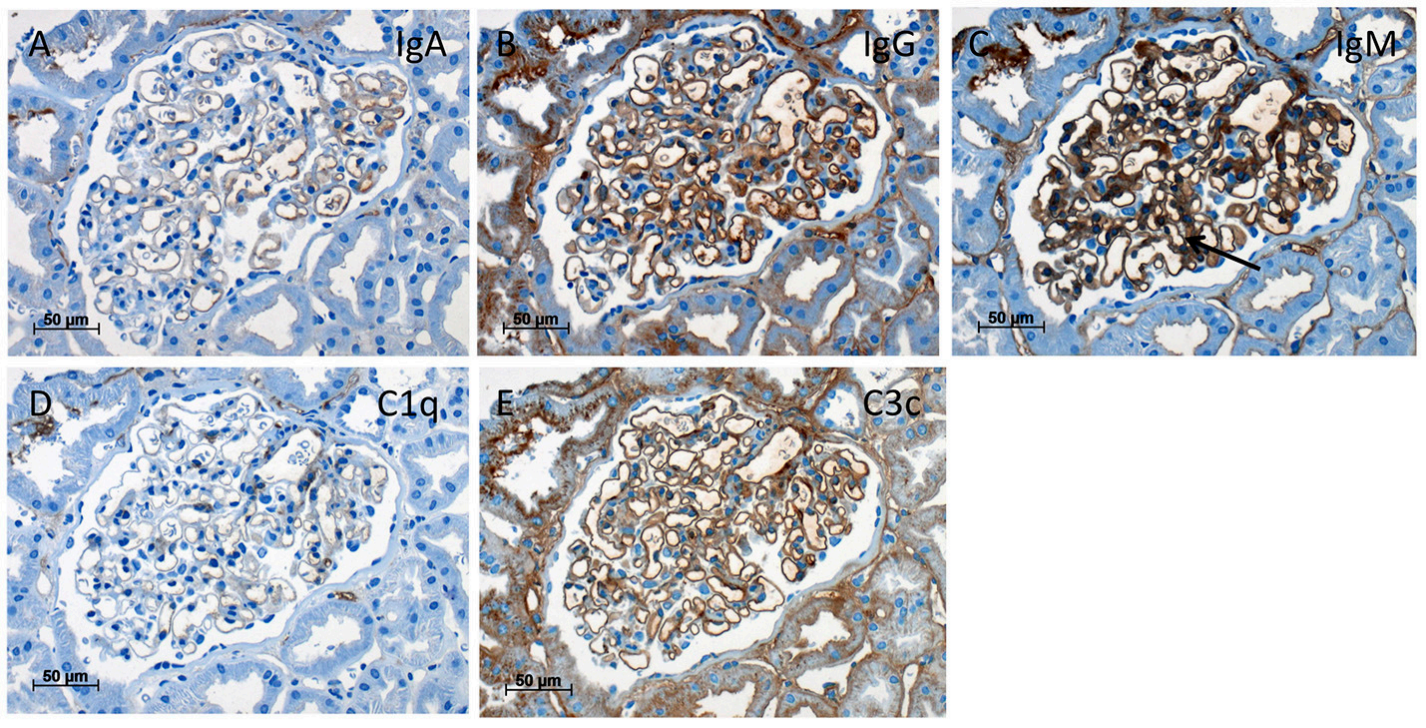
Immunohistochemistry of the kidney biopsy. Immunohistochemistry stainings (magnification x400) with antisera against IgA **(A)**, IgG **(B)**, IgM with mild mesangial staining (**C**, arrow), C1q **(D)**, and C3c **(E)** showed only mild background staining at the endothelial aspect of the capillary loops without deposits suspicious of immune-complex glomerulonephritis.

**Figure 4:**
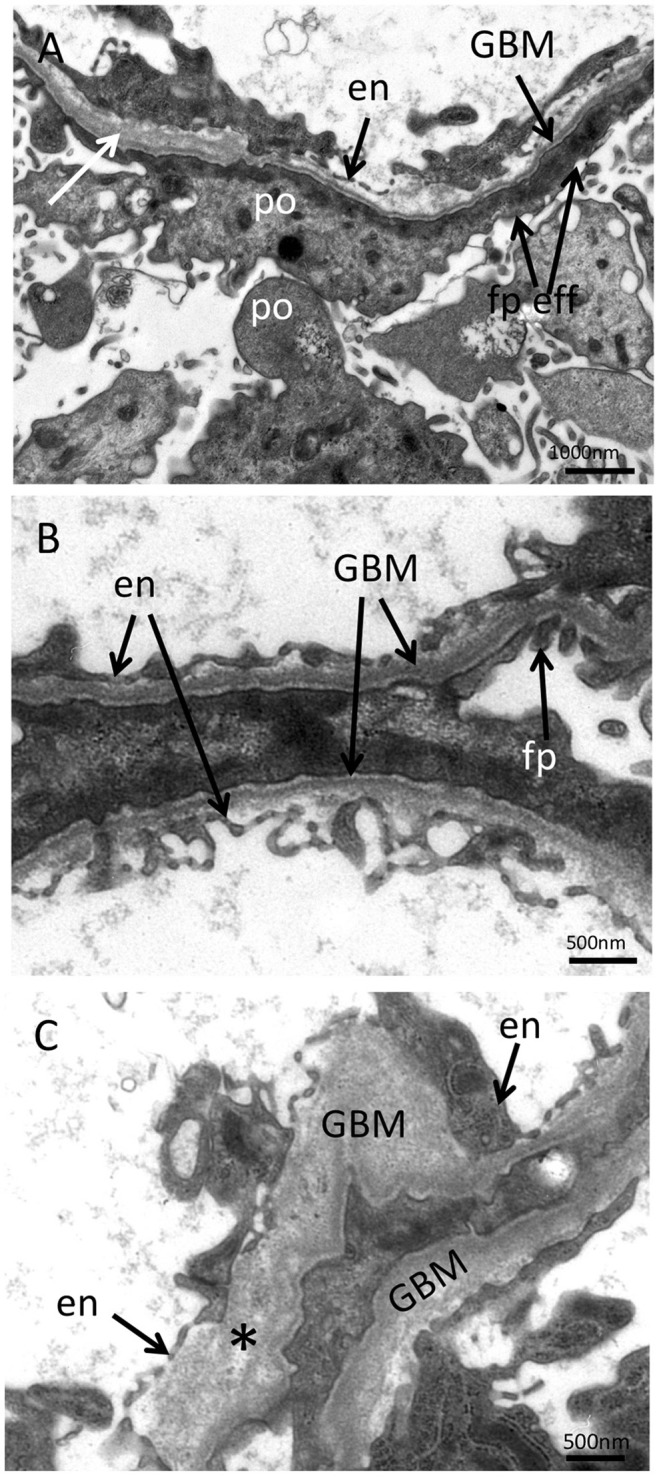
Electron microscopy of the kidney biopsy. **(A)** In the ultrastructural analysis performed at the age of 32, podocytes showed strong foot process effacement with microvillous transformation and thinned glomerular basement membrane (GBM) with mild lamellation (white arrow) compatible with the preexisting diagnosis of primary FSGS (magnification x10,000). In additional ultrastructural analyses performed at the age of 34, segments with thinning and thickening of the GBM (**B**; magnification x16,000) with disrupted basket-woven texture (**C**, asterisk; magnification x16,000) were found. en, endothelium; po, podocyte; fp, foot process; fp eff, podocyte food process effacement.

Light microscopy of the third kidney biopsy revealed renal parenchyma with 11 glomeruli of which four were obliterated (Figure 2A). In one obliterated glomerulus fibrous thickening of the Bowman's capsule was present, indicating fibrous synechia (Figure 2C). Crescents were not identified. The remaining glomeruli were unremarkable (Figure 2D). No segmental sclerosis, extra- or intracapillary proliferation was seen. Patchy tubular atrophy and interstitial fibrosis was present in ~15% (Figures 2E,F) and was accompanied by a mild lymphoplasmacytic interstitial infiltrate. Additionally, multiple foam cell nests were present in the interstitium (Figure 2B). Arterial vessel walls showed mild thickening (Figure 2G) and moderate arteriolar hyalinosis (Figure 2H). In immunohistochemical stainings with antisera against IgA, IgG, IgM, C1q, and C3c only mild mesangial staining for IgM was seen (Figure 3). Furthermore, electron microscopy showed that the GBM (glomerular basement membrane) was moderately thinned with extensive foot process effacement and microvillous transformation of podocytes going in line with the former report of primary FSGS (Figure 4). After an in-patient stay, an increase of MMF to 750 mg/d was recommended. Hypertension and proteinuria were treated with an ACE inhibitor and candesartan.

Two years later, at the age of 34 years, the patient developed moderate inner ear hearing impairment. Additionally, the 6 year old daughter of the index patient was diagnosed with microscopic hematuria. This new clinical symptomatology combined with a conspicuous family history led to a re-examination of electron microscopy of the kidney biopsy performed at the age of 32 years. In further electron microscopic analyses, segments with thinning and thickening of the GBM (170–700 nm) were found. Moreover, changes in the texture of the GBM were seen in some segments with mild lamellation and basket-woven texture. Moderate effacement of the foot processes was present (Figure 4). The re-evaluation of the ultra-thin sections revealed the beforehand described podocyte abnormalities in addition to structural changes of the GBM. Therefore, the changes in GBM structure in the context of the clinical findings and family history were suspicious of hereditary kidney disease and prompted a molecular genetic examination of genes involved in the pathogenesis of AS.

#### Relatives

The daughter of the index patient presented with microscopic hematuria at the age of 6 years as already mentioned above. So far, the son of the index patient does not show any clinical symptoms of a kidney disease at the age of 9 years. Neither the mother nor the father of the index patient developed (chronic) kidney disease until the age of 54 and 58 years, respectively.

### Genetic analysis

Pathogenic variants in *COL4A5* could not be identified. The sequencing of *COL4A3* (NM_000091.4) revealed two missense variants in a compound heterozygous state in the index patient: c.1892G>T, p.Gly631Val (paternally inherited) and c.4981C>T, p.Arg1661Cys (maternally inherited) (Figure 1A, I-1 and I-2). The variants are located in exon 26 and 52, respectively (Figures 1B,D). Each parent is a heterozygous carrier for one variant (Figure 1B). The amino acid at position 631 is highly conserved in evolution (Figure 1C). It is located in the triple helical collagenous domain of the type IV collagen alpha-3 chain (Figure 1D). The variant has not been described in the literature before. The second variant, p.Arg1661Cys, has been described previously and is mostly associated to early development of end-stage renal disease (ESRD) (8, 12, 14). The encoded amino acid is located in the cross-linking C-terminal globular non-collagenous domain (NC1) (14) (Figure 1D). The daughter of the index patient carries the newly discovered variant p.Gly631Val (Figure 1A, III-2). This variant was also present in the father of the index patient (Figure 1A, I-1). The son of the index patient carries the variant p.Arg1661Cys (Figure 1A, III-1).

## Discussion

We identified a novel variant in *COL4A3* combined with a previously described variant in a compound heterozygous state. In the context of the progressive proteinuria and the young age of the index patient the initial diagnosis was erroneously considered to be hereditary FSGS as it was the most probable differential diagnosis. The present study highlights the importance of including AS in the differential diagnosis of FSGS, especially when disease onset is early in life (<25 years of age). In these cases, it would be helpful to start a molecular genetic analysis promptly (i.e., already if mild proteinuria is present).

Several reports have linked variants in *COL4A3* and *COL4A4* to patients with nephrotic-range proteinuria and the histological findings of FSGS (8, 15).

In these cases, FSGS most likely occurs secondary to GBM pathology and therefore represents an independent entity different from primary FSGS with podocytopathy. Histologic diagnosis of FSGS depends on the size of the kidney biopsy as FSGS due to its local nature is prone to sampling error.

Using molecular diagnostics, we identified a novel missense variant in *COL4A3*. The variant p.Gly631Val is assumed to be disease-causing as it segregates within the family and since glycine is important for the spatial organization of the collagen triple helix as it resides at every third position of the collagen type IV chain. Using PolyPhen-2 (http://genetics.bwh.harvard.edu/pph/) and MutationTaster (http://www.mutationtaster.org) to predict possible functional effects, the variant was classified as probably damaging (PolyPhen-2) and disease-causing (MutationTaster). The variant was not found in our in-house database and in the genome Aggregation Database (gnomAD).

The daughter of the index patient, who is a heterozygous carrier of the variant, already demonstrated microscopic hematuria in early childhood (6 years of age). This can be a first sign of thin basement membrane nephropathy (TBMN). However, the father of the index patient, who also carried this variant, showed no signs of (chronic) kidney disease going in line with the wide range of clinical phenotypes reported in individuals with single heterozygous variants (8, 12, 16).

The second variant, p.Arg1661Cys, has been identified both in a heterozygous and compound-heterozygous state in patients with autosomal recessive AS mostly at an early age at diagnosis of 10–18 years (in one case 44 years of age). In one case ESRD was reached at 19 years of age (8, 12). Previous cases presented with nephrotic range proteinuria (2–5 g/d), thickened GBM and foot process effacement. Hearing loss was reported in one of six cases. No ocular disease was reported (8, 12). However, so far, the variant was only reported in heterozygous form to be associated with ESRD (8) (family DUK6585, individual 1). Elsewhere, the variant was reported in a compound-heterozygous state, however, age of renal failure was not indicated (12). This exemplifies the wide range of clinical manifestation of heterozygous variants reaching from familial benign hematuria to AS with development of ESRD. The healthy son and the mother of the index patient carry the same variant and did not develop (chronic) kidney disease so far.

Because both parents and children of the index patient carry a heterozygous variant they should be monitored closely by a (pediatric) nephrologist if hypertension, proteinuria, or renal impairment is present (17). In this case, an ACE inhibitor therapy should be evaluated. Otherwise, in the absence of these symptoms regular examination every 1–2 years should be performed by a primary care physician (17). In patients with TBMN and isolated microscopic hematuria chronic renal failure is rare (16). However, if TBMN is accompanied by proteinuria, it has been associated with an increased risk to late onset FSGS with nephrotic syndrome (18, 19). Furthermore, in clinically affected patients with heterozygous variants in *COL4A3, COL4A4* an increased risk of progression to chronic renal failure was observed in up to 38% and a progression to ESRD was observed in up to 20% (15). Age of ESRD onset was significantly earlier in untreated patients with heterozygous variants in *COL4A3, COL4A4*, and *COL4A5* compared to those treated with blockade of the renin aldosterone system (20). Patients that progress to chronic renal failure and ESRD could have variants in hitherto unknown modifier genes.

The rapid course of disease in one family (DUK6696) with compound heterozygous variants in *COL4A3* (p.Glu131fs^*^151 and p.Gln936^*^) and nephrotic range proteinuria and hematuria with ESRD at 8 to 12 years of age described by Malone et al. is comparable to our family (8). However, the severity and the more rapid course to ESRD in the previous report could be explained by the presence of two loss of function variants whereas our index patient has compound heterozygous missense variants. This is in line with a genotype-phenotype correlation postulated for *COL4A3* as nonsense or larger rearrangement variants lead to a shortening of the protein and are associated with a younger age at renal failure (<20 years) (15, 21). The unusual appearance of AS with nephrotic range proteinuria in our index patient and the cases described by Malone et al. could be additionally modified by variants in FSGS genes that alter the podocyte in collagen IV-related kidney disease and therefore, could explain the high clinical variability. However, Malone et al. was not able to identify variants in genes associated with FSGS in his cohort (8).

Our index patient developed hearing impairment at the age of 34 years. In general, hearing loss seems to occur more often in cases with variants in *COL4A3* as compared to *COL4A4* (8, 12), but is not mandatory. In the study of Malone et al., hearing impairment was only diagnosed in all 8 of 15 individuals with variants in *COL4A3* after genetic testing had already been done (8). Accordingly, in our case otologic examination was not performed at earlier age probably due to the absence of (severe) symptoms.

Several points may have led to the misdiagnosis of hereditary FSGS in our index patient. First, the quality of the kidney biopsy was low. The histological definition of FSGS comprises a broad spectrum of different pathologies together. So far it is not possible to definitely differentiate between subgroups of FSGS based on the morphology. To improve diagnostic accuracy ultrastructural analysis including measurements of the GBM in ultra-thin slices could be helpful to identify GBM defects. In most cases genetic testing has no therapeutic consequence except for the omission of immunosuppressive therapy and therefore avoidance of adverse side effects. Next generation sequencing would allow a much faster and more accurate diagnosis in clinically and genetically heterogeneous cases. Second, nephrotic proteinuria is unusual for AS (10). Third, classical AS symptoms (e.g., hearing impairment) occurred late in the medical history. Fourth, variants in *COL4A3* show a highly variable clinical phenotype. Fifth, as classically men are affected by X-linked inherited AS female patients may be overseen.

Genetic implications are different for patients with autosomal recessive AS and could highlight caution in case of a kidney transplantation. The mode of inheritance is different compared to X-linked AS. A heterozygous (autosomal dominant) affection with microscopic hematuria and TBMN warrants a restrained selection as kidney donor. This is especially important if blood pressure is high or proteinuria is present (17). Therefore, the newly discovered p.Gly631Val variant with the clinical presentation of microscopic hematuria in the daughter indicates caution for selection as kidney donor.

In patients with AS and TBMN ACE inhibitors are recommended by the expert guidelines for the treatment of hypertension and proteinuria especially in individuals with genetic variants (17). Angiotensin-receptor blockers and aldosterone inhibitors could show additional effects on the reduction of proteinuria (22). To date, new insights for the treatment of AS are expected from the EARLY PRO-TECT Alport trail that is evaluating the effect of renin-angiotensin blockade on proteinuria and renal failure progression (23).

Some limitations of the present study have to be stated. We performed only targeted sequencing of *COL4A3* and *COL4A5* as this study was already performed several years ago. Examination of disease modifiers for collagen IV that could explain the high variability of phenotypes described before were not performed (8). Today panel diagnostics, whole exome or whole genome sequencing would be done in such a case. Also, we did not examine other genes or modifiers related to FSGS, which should be included in further studies to improve the understanding of the high variability of the clinical phenotype. Due to the absence of cryopreserved tissue of the kidney biopsies, it was not possible to perform immunofluorescence analysis with antibodies specific for alpha-3,4,5 subunits of collagen IV complex in the GBM and therefore directly document collagen dysfunction. Unfortunately, electron microscopic images for a re-examination were only available from the last kidney biopsy.

## Conclusion

We were able to show that a novel variant combined with a previously described variant in *COL4A3* in compound heterozygous state can lead to a phenotype that was erroneously associated with hereditary FSGS. Finally, our study exemplifies the importance of molecular examination in the diagnosis of the renal phenotype to improve diagnostic accuracy and avoid unnecessary inefficient treatment with immunosuppression.

## Author contributions

MB and JH, analyzed and interpreted the patient data regarding the genetic and clinical findings and wrote the manuscript. MB-H conducted the histological examination of the third kidney biopsy, compiled histological, and electron microscopy figures and contributed to the writing of the manuscript. H-GK, DW, and JH performed the molecular diagnostics. CK, LR, UH, and CS conducted in-patient treatment. RG, RS, KR, P-MH, CK, LR, UH, and CS contributed important intellectual content during manuscript drafting and revision. All authors accept accountability for the overall work by ensuring that questions pertaining to the accuracy or integrity of any portion of the work are appropriately investigated and resolved. Text revision was performed by all authors. All authors read and approved the final manuscript.

### Conflict of interest statement

The authors declare that the research was conducted in the absence of any commercial or financial relationships that could be construed as a potential conflict of interest.

## Abbreviations

ACE: angiotensin-converting enzyme
AS: Alport syndrome
ESRD: end-stage renal disease
FSGS: focal segmental glomerulosclerosis
GBM: glomerular basement membrane
MMF: mycophenolate mofetil
NC1: C-terminal globular non-collagenous domain
SRNS: steroid-resistant nephrotic syndrome
TBMN: thin basement membrane nephropathy.

## References

[1] Benoit G Machuca E Antignac C. Hereditary nephrotic syndrome: a systematic approach for genetic testing and a review of associated podocyte gene mutations. Pediatr Nephrol. (2010) 25:1621–32. 10.1007/s00467-010-1495-0 20333530 PMC2908444

[2] Caridi G Trivelli A Sanna-Cherchi S Perfumo F Ghiggeri GM. Familial forms of nephrotic syndrome. Pediatr Nephrol. (2010) 25:241–52. 10.1007/s00467-008-1051-3 19066979 PMC6904408

[3] Sadowski CE Lovric S Ashraf S Pabst WL Gee HY Kohl S. A single-gene cause in 29.5% of cases of steroid-resistant nephrotic syndrome. J Am Soc Nephrol. (2015) 26:1279–89. 10.1681/ASN.2014050489 25349199 PMC4446877

[4] D'agati VD Kaskel FJ Falk RJ. Focal segmental glomerulosclerosis. N Engl J Med. (2011) 365:2398–411. 10.1056/NEJMra1106556 22187987

[5] Warejko JK Tan W Daga A Schapiro D Lawson JA Shril S. (2018). Whole exome sequencing of patients with steroid-resistant nephrotic syndrome. Clin J Am Soc Nephrol. 13:53–62. 10.2215/CJN.04120417 29127259 PMC5753307

[6] Korbet SM. Treatment of primary FSGS in adults. J Am Soc Nephrol. (2012) 23:1769–76. 10.1681/ASN.2012040389 22997260

[7] Ponticelli C Graziani G. Current and emerging treatments for idiopathic focal and segmental glomerulosclerosis in adults. Expert Rev Clin Immunol. (2013) 9:251–61. 10.1586/eci.12.109 23445199

[8] Malone AF Phelan PJ Hall G Cetincelik U Homstad A Alonso AS. Rare hereditary COL4A3/COL4A4 variants may be mistaken for familial focal segmental glomerulosclerosis. Kidney Int. (2014) 86:1253–9. 10.1038/ki.2014.305 25229338 PMC4245465

[9] Kashtan CE. Alport syndrome. An inherited disorder of renal, ocular, and cochlear basement membranes. Medicine (1999) 78:338–60. 10.1097/00005792-199909000-00005 10499074

[10] Hebert LA Parikh S Prosek J Nadasdy T Rovin BH. Differential diagnosis of glomerular disease: a systematic and inclusive approach. Am J Nephrol. (2013) 38:253–66. 10.1159/000354390 24052039 PMC3842189

[11] Pescucci C Mari F Longo I Vogiatzi P Caselli R Scala E Autosomal-dominant Alport syndrome: natural history of a disease due to COL4A3 or COL4A4 gene. Kidney Int. (2004) 65:1598–603. 10.1111/j.1523-1755.2004.00560.x 15086897

[12] Storey H Savige J Sivakumar V Abbs S Flinter FA. COL4A3/COL4A4 mutations and features in individuals with autosomal recessive Alport syndrome. J Am Soc Nephrol. (2013) 24:1945–54. 10.1681/ASN.2012100985 24052634 PMC3839543

[13] Hu B Jin J Guo AY Zhang H Luo J Gao G. GSDS 2.0: an upgraded gene feature visualization server. Bioinformatics (2015) 31:1296–7. 10.1093/bioinformatics/btu817 25504850 PMC4393523

[14] Heidet L Arrondel C Forestier L Cohen-Solal L Mollet G Gutierrez B. Structure of the human type IV collagen gene COL4A3 and mutations in autosomal Alport syndrome. J Am Soc Nephrol. (2001) 12:97–106. 11134255 10.1681/ASN.V12197

[15] Voskarides K Damianou L Neocleous V Zouvani I Christodoulidou S Hadjiconstantinou V. COL4A3/COL4A4 mutations producing focal segmental glomerulosclerosis and renal failure in thin basement membrane nephropathy. J Am Soc Nephrol. (2007) 18:3004–16. 10.1681/ASN.2007040444 17942953

[16] Savige J Rana K Tonna S Buzza M Dagher H Wang YY. Thin basement membrane nephropathy. Kidney Int. (2003) 64:1169–78. 10.1046/j.1523-1755.2003.00234.x 12969134

[17] Savige J Gregory M Gross O Kashtan C Ding J Flinter F. Expert guidelines for the management of Alport syndrome and thin basement membrane nephropathy. J Am Soc Nephrol. (2013) 24:364–75. 10.1681/ASN.2012020148 23349312

[18] Van Paassen P Van Breda Vriesman PJ Van Rie H Tervaert JW. Signs and symptoms of thin basement membrane nephropathy: a prospective regional study on primary glomerular disease-The Limburg Renal Registry. Kidney Int. (2004) 66:909–13. 10.1111/j.1523-1755.2004.00835.x 15327380

[19] Deltas C. Thin basement membrane nephropathy: is there genetic predisposition to more severe disease? Pediatr Nephrol. (2009) 24:877–9. 10.1007/s00467-008-1042-4 19018577 PMC7811520

[20] Temme J Peters F Lange K Pirson Y Heidet L Torra R. Incidence of renal failure and nephroprotection by RAAS inhibition in heterozygous carriers of X-chromosomal and autosomal recessive Alport mutations. Kidney Int. (2012) 81:779–83. 10.1038/ki.2011.452 22237748

[21] Savige J Storey H Il Cheong H Gyung Kang H Park E Hilbert P. X-Linked and autosomal recessive alport syndrome: pathogenic variant features and further genotype-phenotype correlations. PLoS ONE (2016) 11:e0161802. 10.1371/journal.pone.0161802 27627812 PMC5023110

[22] Kaito H Nozu K Iijima K Nakanishi K Yoshiya K Kanda K. The effect of aldosterone blockade in patients with alport syndrome. Pediatr Nephrol. (2006) 21:1824–9. 10.1007/s00467-006-0270-8 17039334

[23] Gross O Friede T Hilgers R Gorlitz A Gavenis K Ahmed R. Safety and efficacy of the ACE-inhibitor ramipril in Alport syndrome: the double-blind, randomized, placebo-controlled, multicenter phase III Early Pro-Tect Alport trial in pediatric patients. ISRN Pediatr. (2012) 2012:436046. 10.5402/2012/436046 22811928 PMC3395192

